# Supplementary material for: EMG-projected MEG high-resolution source imaging of human motor execution: Brain-muscle coupling above movement frequencies
Source: Imaging Neurosci (Camb). 2024 Jan 9;2:imag-2-00056. doi: 10.1162/imag_a_00056 (PMC11403128; doi:10.1162/imag_a_00056)
Supplement: Supplementary Material [file imag_a_00056-supp.pdf]

Supplementary Materials

Additional median-nerve test for clinical patients:

MEG recordings were conducted for the three clinical patients with lesions near the central sulcus as they underwent median-nerve stimulation using a bipolar constant current stimulator. The stimuli were square-wave electric pulses (0.2 ms duration) delivered at about 1 Hz (ISI: 800 ms to 1200 ms). The intensity of the stimulation was adjusted until robust thumb twitches were observed. Magnetic fields evoked by median-nerve stimulation were measured using our MEGIN/Neuromag whole-head Vectorview™ MEG system. EOG electrodes were used to detect eye blinks and eye movements. An interval of 500 ms post-stimulus was recorded, using 300 ms pre-stimulus data for constructing the noise covariance matrix for pre-whitening. Data were sampled at 1000 Hz and run through a band-pass filter with 0.1-333 Hz Hz cut-off and through a notch filter (58-62 Hz) to remove 60Hz power-line noise. One hundred artifact-free responses were averaged with respect to the stimulus trigger to increase the SNR.

The MEG source imaging of the trial-averaged median-nerve responses was obtained for the M20 component (~20ms post-stimulus) using our Fast-VESTAL source imaging program (Huang et al., 2014a; 2016; Zheng et al., 2021). The focus was the localization of the primary somatosensory (S1) cortex.

Additional figures for supporting the main text:

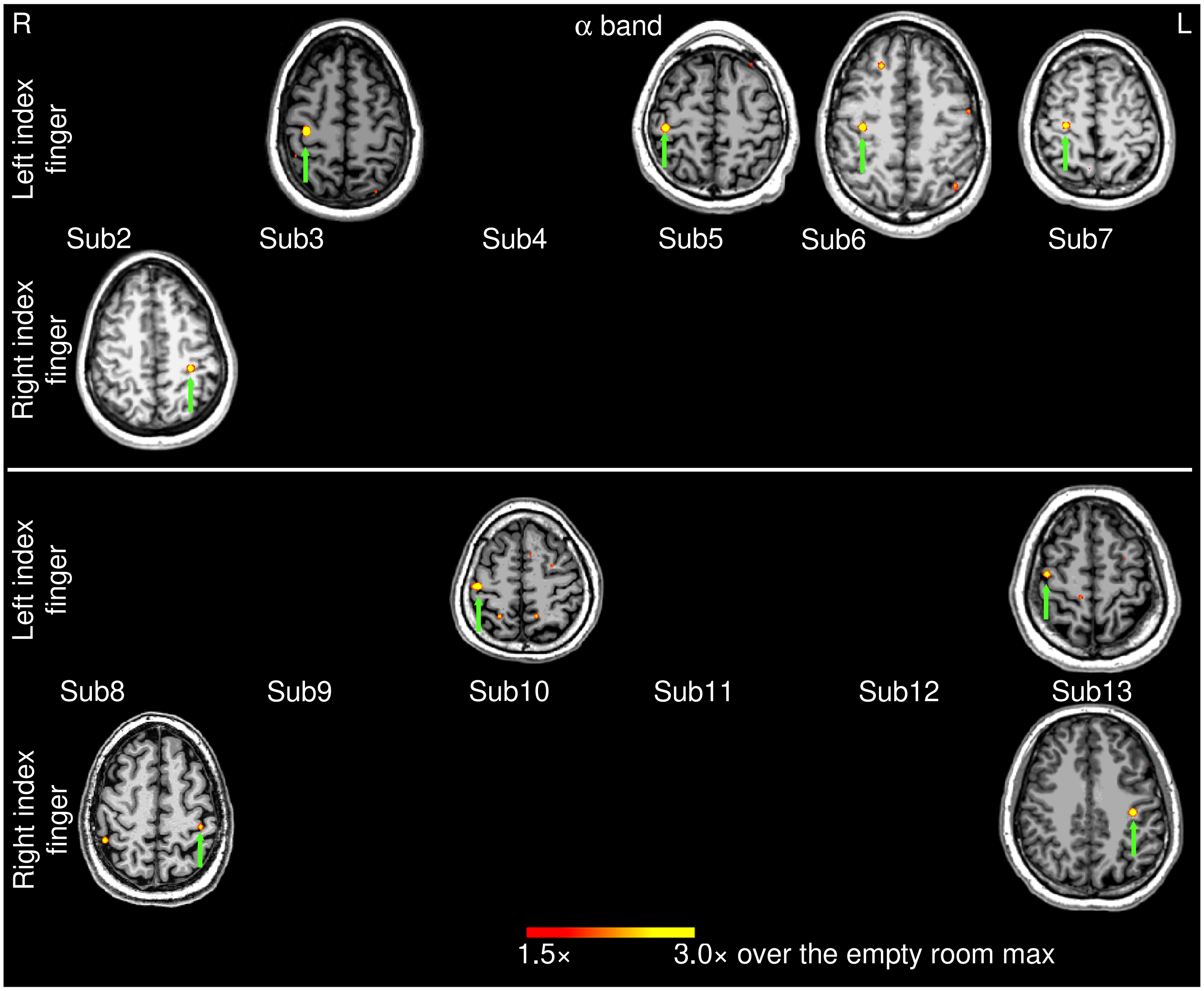

Figure S1: Movement-related alpha-band EMG-projected MEG source images for the remaining 12 healthy subjects. Significant primary motor cortex sources (green arrows) contralateral to left or right self-paced (~1 min) index finger movements. The color bar shows the activity threshold at 1.5× of the empty room maximum value, and saturation level at 3.0×. In 6 cases, contralateral primary motor source activity at or above the threshold was not observed. Sub = subject

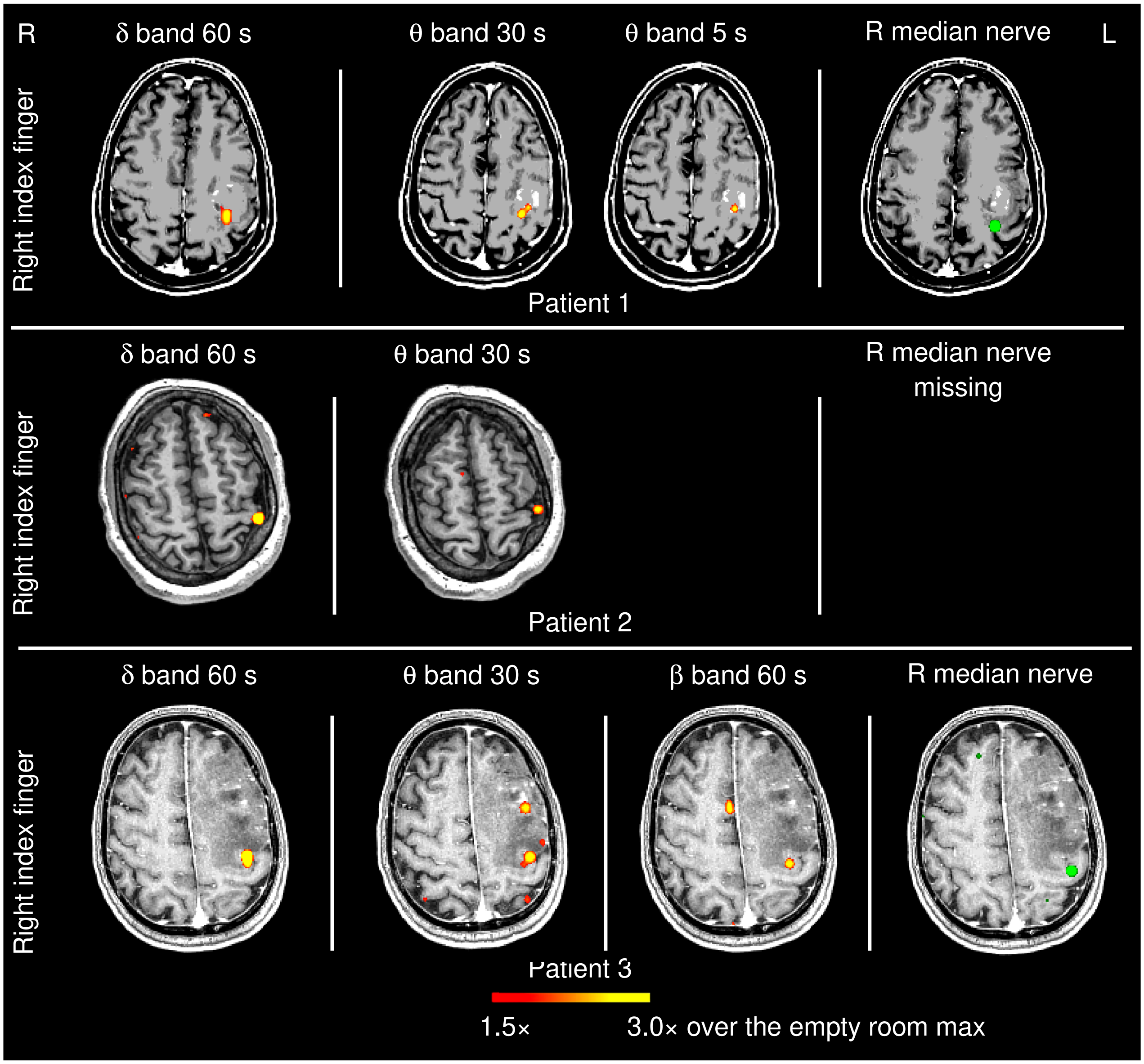

Figure S2: This figure provides supplementary results to Figure 5 in the main text: EMG-projected MEG source images of M1cortex in the *affected* left hemispheres due to right finger movement (affected hand) from the three clinical patients in theta and delta bands. Delta-band M1 cortical activity (1 min EEG recordings) in the damaged hemisphere was localized in all three patients. In the theta band, M1 was localized in 30 sec and 5 sec time windows in Patient 1, but only the 30 sec time window in Patient 2 and Patient 3. In beta band, M1 was localized in 60-sec time window only in Patient 3. In addition, the right median nerve responses were localized to the primary somatosensory cortex of the damaged left hemispheres in Patients 1 and 3, but was missing in Patient 2.

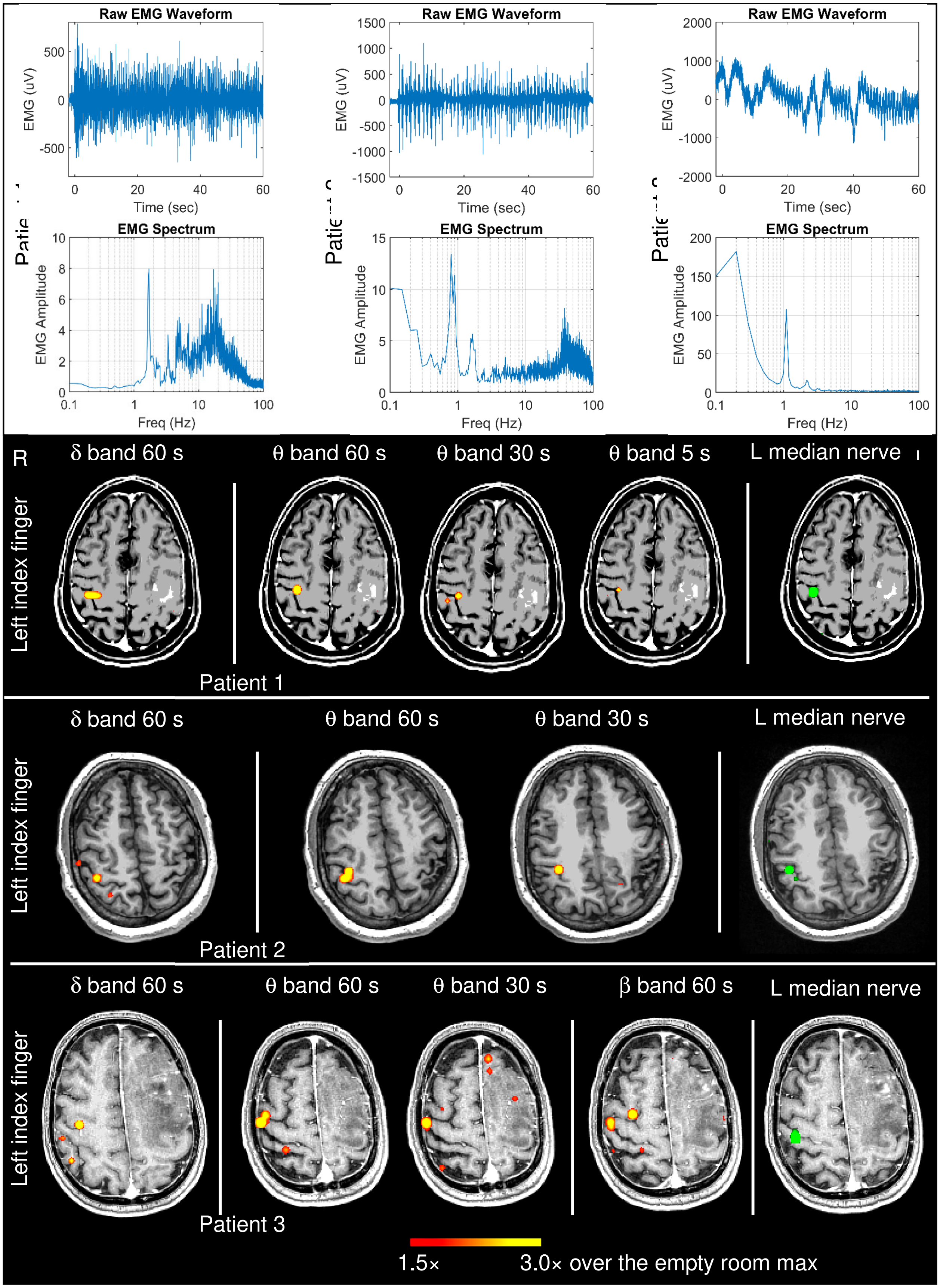

Raw EMG Waveform
EMG (uV)
Time (sec)
EMG Spectrum
EMG Amplitude
Freq (Hz)
R
δ band 60 s
θ band 60 s
θ band 30 s
θ band 5 s
L median nerve
Left index finger
Patient 1
Patient 2
Patient 3
β band 60 s
1.5×
3.0× over the empty room max

Figure S3: EMG-projected MEG source images of M1cortex in the *unaffected* right hemispheres due to left finger movements (*unaffected* hand) from the three clinical patients. Top Panel: the EMG waveforms and spectra from the left index finger movement (unaffected hand). Bottom Panel: EMG-projected MEG source images of M1 in the unaffected right hemispheres due to left finger movement from the three patients. Delta-band M1 activity (1 min EEG recordings) was localized in the unaffected hemisphere in all three patients. In Patient 1, theta-band M1 cortex activity was localized in the 60 sec, 30 sec and 5 sec time windows. In Patients 2 and 3, theta-band M1 activity was localized only in the 60 sec and 30 sec time windows. In beta band, M1 was localized in 60-sec time window only in Patient 3. In addition, the left median nerve responses (unaffected hand) were localized to the primary somatosensory cortex of the unaffected right hemispheres in all three patients.
